# Analysis of the complete mitochondrial genomes of two dwarf honeybee species, *Apis florea* and *Apis andreniformis* (Insecta: Hymenoptera: Apidae), in Thailand

**DOI:** 10.1080/23802359.2018.1450672

**Published:** 2018-03-14

**Authors:** Jun-ichi Takahashi, Sureerat Deowanish, Hisashi Okuyama

**Affiliations:** a Faculty of Life Sciences, Kyoto Sangyo University, Kyoto, Japan;; b Department of Biology, Chulalongkorn University, Bangkok, Thailand

**Keywords:** Mitochondrial DNA, *Micrapis*, dwarf honey bee, next generation sequencing

## Abstract

The dwarf honeybees *Apis florea* and *Apis andreniformis* inhabit the bush and forests of continental Asia and north Africa and some islands of Sundaland and the Philippines. We analysed, for the first time, the complete mitochondrial genomes of two dwarf honeybee species from Thailand using next-generation sequencing. Each mitochondrial genome was a circular and approximately 17 kbp molecule that included 13 protein-coding genes (PCGs), 22 tRNA genes, and two ribosomal RNA genes, along with one A + T-rich control region, besides three tRNA-*Ser* (*AGN*) repeats. The AT content values of the mitochondrial genomes of *A. florea* and *A. andreniformis* were 86.28% and 85.73%, respectively. The 1150 mutation sites in 13 PCGs differing between *A. florea* and *A. andreniformis* in Thailand were evenly distributed throughout their mitochondrial genomes. The phylogenetic relationship, inferred using 13 PCGs, was consistent with that reported in previous studies, which predicted a sister relationship between *A. florea* and *A. andreniformis*.

The dwarf honeybee group (subgenus *Micrapis*) is distributed in the bush and forests of continental Asia to north Africa, as well as parts of Sundaland and the Philippines (Otis 1996). The dwarf honeybee is divided into red and black types on the basis of the abdominal colour of adult workers (Maa 1953; Sakagami et al. 1980; Ruttner 1988; Engels 1999). The black dwarf honeybee was classified as a variant or subspecies of the red dwarf honeybee *Apis florea* until it was re-established by Maa (1953). Although the classification of the black dwarf honeybee remained controversial, it was subsequently reconfirmed that *A. andreniformis* is a valid species, different from *Apis florea*, on the basis of morphological differences in the adult male genitalia (Oldroyd and Wongsiri 2006). Morphological studies also reported that the adult males of the dwarf honeybees *A. florea* and *A. andreniformis* differ in the shape and size of the bifurcated basitarsus of the hind leg and the structure of the endophallus (Oldroyd and Wongsiri 2006). Morphometric analyses between *A. florea* and *A. andreniformis* revealed differences in variables, including the jugal-vannal ratio of the hindwing and cubital index of adult workers (Hepburn and Radloff 2011). Molecular phylogenetic analyses of partial mitochondrial DNA sequences also indicated that *A. andreniformis* is a sister taxon and a separate species from *A. florea* (Tanaka et al. 2001; Arias and Sheppard 2005; Lo et al. 2010). The red dwarf honeybee *A. florea* inhabits forests from lowlands to mountainous regions, widely distributed from Iran to southwestern China, islands of Indonesia, and northern Africa. Meanwhile, the black dwarf honeybee *A. andreniformis* is only found in Vietnam, Thailand, Laos, China, and India; and the islands of Borneo, Indonesia, and Palawan. The sympatric distributions of *A. florea* and *A. andreniformis* exist mainly in Thailand in South East Asia (Otis 1996; Hepburn and Radloff 2011).

Phylogenetic analysis of dwarf honeybees has suggested the presence of two species, *A. florea* and *A. andreniformis*, and the dwarf honeybee group may contain several subspecies (Hepburn and Radloff 2011). Analysis of the dwarf honeybee using RFLP of mitochondrial DNA and microsatellite DNA has shown that haplotype diversity of *A. andreniformis* was a quarter of that of *A. florea*, and also lower than that of other honeybee species (Takahashi et al. 2008; Hepburn and Radloff 2011). The genetic variation of the mitochondrial DNA sequence in the red dwarf honeybee *A. florea* is lower than that of the sympatric honeybees *Apis cerana* and *Apis dorsata* (Rattanawannee et al. 2007; Takahashi et al. 2017). The haplotype numbers of the mitochondrial COI, and *Cytb* and 16S genes in *A. andreniformis* were less than those of the sympatric honeybee *A. florea* (Takahashi 2006). The genetic diversity of *A. andreniformis* provides little information because the mitochondrial DNA sequence data have a few sequences registered in the BOLD (The Barcode of Life Data) systems at present. These results suggest that *A. andreniformis* has a lower genetic diversity than that of other Asian honeybee species.

Dwarf honeybees require several habitat areas because they change their nesting site every season in Thailand (Oldroyd and Wongsiri 2006). Thus, these honeybees are especially affected by deforestation. The possibility of genetic fragmentation and extinction risk is higher in dwarf honeybees because they have low mobility, which exacerbates the effects of habitat fragmentation. The knowledge base of the genetic diversity and classification of dwarf honeybees for their conservation has been inadequate thus far. Here, we report the first analysis of the full-length sequence of mitochondrial DNA of the dwarf honeybees *A. florea* and *A. andreniformis* in Thailand.

We collected adult worker bees of *A. florea* and *A. andreniformis* from flowers in the campus of Chulalongkorn University in Thailand (13°44′31.2″N, 100°31′40.5″E). The collected worker bees were immediately placed in 99% ethanol for mitochondrial DNA analysis (these specimens were stored in the National Museum of Nature and Science, Japan, accession number: NSMT-I-HYM 75333 and NSMT-I-HYM 75334). Genomic DNA was extracted from the thoracic muscle tissue using standard phenol/chloroform methods. Genomic DNA was sequenced using NextSeq 500 technology (Illumina, San Diego, CA). The complete mitochondrial genomes of various dwarf honeybees were used as reference sequences (Wang et al. 2013, 2015; Yang et al. 2017). The resulting reads were assembled and annotated using the MITOS web server (Bernt et al. 2013) and MEGA6 (Tamura et al. 2013). Phylogenetic analysis was performed using TREEFINDER v.2011 (Jobb 2015) based on the nucleotide sequences of the 13 protein-coding genes (PCGs) from the complete mitochondrial genome sequences of *Apis* species used in this study, as well as those present in DNA Data Base of Japan.

The mitochondrial genomes of *A. florea* and *A. andreniformis* from Bangkok Metropolitan Region, Thailand, were both closed loops of 17,693 and 16,694 bp, respectively (AP018491 and AP018490). The mitochondrial genomes of *A. florea* and *A. andreniformis* represent a typical hymenopteran mitochondrial genome (Figure 1). In the mitochondrial genomes of both *A. florea* and *A. andreniformis*, the heavy (H) strand encoded nine PCGs and 14 tRNA genes, while the light (L) strand encoded four PCGs, eight tRNA genes, and two rRNA genes22 tRNA genes, along with one A + T-rich control region, besides three tRNA-*Ser*(*AGN*) repeats (Figure 1). The average AT content values were 86.28% and 85.73%, respectively. The genes *ATP8* and *ATP6* of *A. florea* and *A. andreniformis* shared 19 nucleotides. All the tRNA genes possessed cloverleaf secondary structures except for *tRNA*-*Ser* (*S1*) and *tRNA*-*Glu* (*E*).

**Figure 1.**
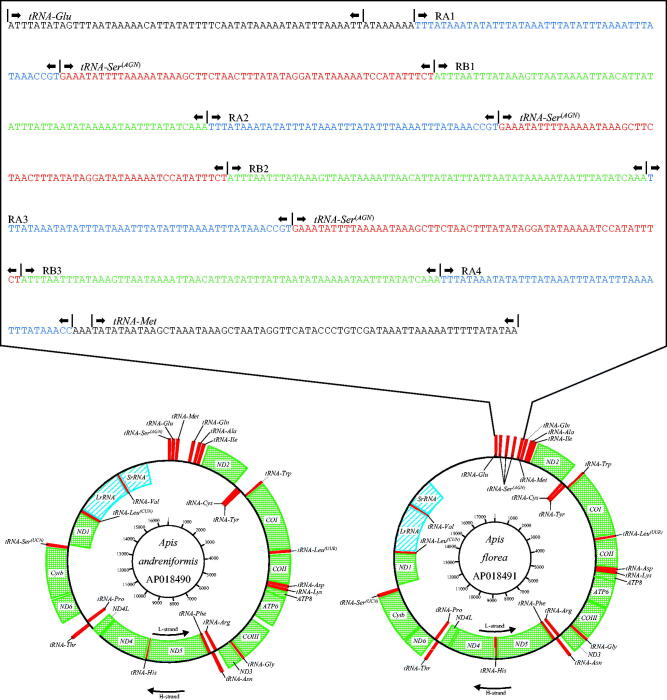
Physical map of the mitochondrial genome of *Apis florea* and *Apis andreniformis*. Genes illustrated on the outside of the main circle are encoded on the heavy (H) strand; genes on the inside of the circle are encoded on the light (L) strand. The 13 protein-coding genes are labelled in Grid line (green), 22 tRNA genes are labelled in square (red), and LrRNA and SrRNA genes are labelled in oblique line (blue).

Phylogenetic analysis was performed using the sequences of 13 mitochondrial PCGs, as well as those of 10 closely related taxa (Figure 2). The phylogenetic analysis suggested that *A. florea* from Thailand is most closely related to Chinese *A. florea*, and that *A. florea* and *A. andreniformis* are sister species. This result is consistent with a published phylogenetic analysis inferred from the mitochondrial DNA of *A. florea* and *A. andreniformis* (Takahashi 2006). The results also clearly showed that *A. florea* and *A. andreniformis* are valid species, but the classification of subspecies and genetic diversity in dwarf honeybees remain to be elucidated. The complete sequence of the mitochondrial genome of dwarf honeybees provides additional genetic information for conservation genetics studies, and will aid the definition of the population genetic structure and subspecies of dwarf honeybees.

**Figure 2.**
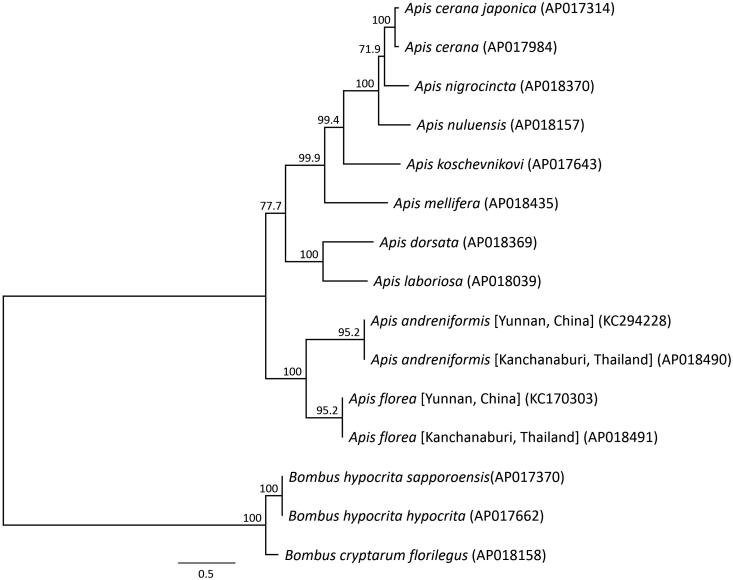
Phylogenetic relationships (maximum likelihood) of species in the genus *Apis* (Hymenoptera) based on the nucleotide sequence of the 13 protein-coding genes in the mitochondrial genome. These sequences were separated by codon positions and for each partition, optimal models of sequence evolution were used in the maximum likelihood method using TREEFINDER, based on the corrected Akaike information criterion. Numbers next to each node represent percentage bootstrap values based on 1000 replications. *Bombus hypocrita* was used as an outgroup. Alphanumeric terms in parentheses indicate GenBank accession numbers.
